# Excision of the Cervix Uteri for Corroding Ulcers

**Published:** 1856-04

**Authors:** Laurence Turnbull

**Affiliations:** Philadelphia


[*Ranking's Abstract.*

---

*Excission of the Cervix Uteri for Corroding Ulcer. Recovery.* By Laurence Turnbull, M. D., Philadelphia.

Martha H., aged twenty-five years, unmarried, has never enjoyed good health, and has always been irregular at her menstrual periods. She had a severe attack of dysentery in 1852, and upon recovery suffered with constant pain in the lower part of the abdomen and region of the uterus, with a sensation of weight and leucorrhœal discharge. She placed herself under the care of a physician who treated her for prolapsus uteri, by means of rest, injections, &c., and an abdominal supporter. She remained under his charge for one month without benefit, the supporter increasing her pain so much that she was unable to wear it. Obtaining no relief, she applied to a second physician, who examined her by touch, and ordered strong injections of nitrate of silver, with some internal remedies, which she employed, but with no amelioration of the pain from which she suffered.

In May, 1853, she came under my care; at that time her menses were slight, and irregular, with occasional vicarious discharges of blood from the stomach; her pain was chiefly in the region of the womb, passing down the limbs: skin pale and anemic, appetite poor, and bowels constipated. I prescribed iron as a tonic, with a gentle aperient, and narcotics to relieve the pain, but made no examination, she not being willing. She continued the use of these medicines for several months, her general health becoming somewhat improved, but still her pain continued.

Towards the end of 1853 she suffered so much, and was so harassed by it, that she reluctantly consented to a more careful examination by the touch and speculum, which brought to view a gray colored ulcer pentrating the neck of the os uteri, and causing it to turn towards the rectum, the neck being also increased in volume.

The treatment pursued was rest, cupping over the sacrum, leeches to the low part of the abdomen with an occasional blister, dressed with mercurial ointment, and the internal use of small doses of calomel, with solid nitrate of silver applied to the ulcer; mucillaginous baths and anodyne injections were also used.

By these means she was made much more comfortable, but the ulcer would not heal; still she was able to engage in her usual occupation.

In January, 1855, I was again called to visit her in haste, on account of an excessive hemorrhage which had taken place from her stomach. This discharge was of more frequent occurrence, also, than before, her menstrual discharge having almost entirely ceased. Fearing the loss of so much blood I prescribed small doses of acetate of lead and opium, which seemed to moderate the flow of blood and diminish her pain.

I now gave her small doses of bichloride of mercury until it produced salivation, and touched the ulcer with the proto-nitrate of mercury, which, however, produced no permanent benefit. After the effects of the mercury had passed away, I placed her upon the internal use of large doses of extractof conium in combination with the iodide of potassum, and changed the local application to the ulcer, but with little advantage to my patient, as she was relieved only for the time, the pain returning with such violence all down her limbs and side as to render her unable to walk, so that she was obliged to give up her work.

She now desired to know if it were not possible to do something more for her, for she remarked "she was not able to bear the pain which she suffered." I told her the only means left was the removal of the neck of the womb by the knife; she said she was perfectly willing.

Having friends and relations, I gave her time for consideration and consultation, and fully explained the uncertain results and difficulties of the operation; she still urged that her life was a burthen to her, and she was willing to run the risk of the operation for the chance of a cure.

On May 21, 1855, I proceeded to operate in the following manner, assisted by my friend Dr. Hewson Bache. Placing the patient on the edge of a bed resting on her knees in the position recommended by Dr. Sims for vesico-vaginal fistula, ether was administered to her, and a metal speculum was placed in the rectal portion of the vagina with one on each side, so as to expose fully the neck of the womb.

Having cleansed the parts by means of a pallet of cotton, the double pincers of Museux were carried close up to the os tincæ, which I seized from before backwards as high as possible; I then commenced a slow and sustained traction until the os was brought to the exterior orifice; (being under the influence of the ether, this gave her little pain); then with the curved knife of Jobert cutting on its concave edge, and holding the pincers with my left hand, I cut through the healthy tissue slowly from left to right, until all the ulcerated and enlarged portions were removed, making a concave even cut of the neck of the womb.

The hemorrhage was very abundant, and poured out of the vagina in a stream for some time; this was checked by plugging the vagina with pieces of old muslin, which were secured by a T bandage. She was then placed upon her side and directed to take one grain of opium every two hours, with iced gum water as drink.

In the afternoon she suffered from severe nervous spasms, which were relieved by the use of sulphuric ether and opium.

May 22.—Was called at four o'clock, A. M., to relieve the bladder and remove the tampon; had taken twelve grains of opium through the night, was restless and still suffered pain. Skin dry, pulse 84, syringed the parts with flaxseed tea, and directed opium to be continued with oat meal gruel as diet.

May 23.—Pain less, slept without the opium, pulse 83, tongue furred. Ordered pil hydr. gr. iij., pulv. opii. gr. j. *m* ft. pilula No. 1. To be taken at night, and followed by ol. ricini in the morning. She continued to suffer a good deal of pain, and required daily attendance until July, when she had a severe attack of convulsions which lasted nearly twelve hours. Towards the end of July she was able to be removed to the country, where she gradually improved, and returned in August, looking and feeling well, with a good color and appetite, and quite able to resume her usual occupation in a cotton mill.

The womb looked healthy, the parts had healed, and menstruation had returned, with no hemorrhage from the stomach.

In the above case every means were employed to relieve previous to the operation, and all had failed.

She had taken in large doses, opium, morphia, ether, chloroform, and many of the antispasmodics; mercury, arsenic and iodine had also been administered internally, so as to produce their specific effects, but without benefit; various local applications had been tried in vain, such as the salts of lime, lead, silver, copper, solution of soot, nitric acid and nitrite of mercury, and the actual cautery when every other application failed in relieving her.

The pain was that of cancer, being "gnawing," and so intense that she was perfectly willing to take any thing, or do any thing to be relieved, and therefore I considered myself perfectly justified in trying the effect of an operation before the whole system became involved.

January 25, 1856.—Martha called to see me; she still suffers some pain at certain periods, but has gained several pounds in weight, and is able to live with much more comfort than before the operation.

In the microscopic examination of the specimen removed by Dr. Da Costa of this city, nothing was found but the normal cells hypertrophied, and fibro-plastic cells.—*Medical Examiner.*

*Phantom Tumors.* By John S. Plummer, M. D. Richmond, Indiana.

These evanescent swellings in the abdomen, have rarely been noticed in our medical publications. And, so far as my acquaintance with these mimic tumors has extended, their causes have been so apparent, that I have not heretofore considered the cases which it has fallen to my lot to observe, as worthy of a record in our periodicals.

But the importance which has recently been attached to them in England, and perhaps correctly, induces me now, to broach the subject for the first time, so far as I am informed, in this country.

In the diagnosis of abdominal tumors, it is averred, that it is sometimes "almost impossible to decide whether or not any tumor does exist. The signs are present one day, entirely absent on another, then present again, in a most perplexing manner." So closely, it is said, does these swellings simulate real tumors, that Dr. Bright tells us, that a surgeon, under the impression that the tumor was an enlarged ovary, proceeded to operate. The incision being made, "no tumor whatever, could be found, and the operator was obliged to desist." After the woman's recovery from the effects of the operation, the tumor reäppeared.

The same physician thinks it even possible that some of the reported cases of disappearance of ovarian cysts, may be set down as instances of erroneous diagnosis, originating in these deceptive swellings. The partial "hysterical distention" of the intestines, may remain, says he, for days or weeks at a time, giving completely the forms of tumors, sometimes with indistinct fluctuation, from fluid feces; and at other times, presenting the characters of a solid tumor, from large accumulations of hardened feculent matter.

These seeming tumors are thought to be more frequent on the right side, than the left, (explainable perhaps, by anatomical considerations,) and to occur oftener in early adult life, than at other periods; and they exist in young men, as well as in females.

"Tumors resulting from accumulation of inflammatory products," "cases of typhlitis" and "tumors springing from the kidney or abscesses in that organ," are the affections for which these fictitious ones are most likely to be mistaken.

Drs. Bright, Addison, and Gull appear to have been the earliest to give notoriety to these capricious enlargements; and to Dr. Gull is ascribed the invention of the happy and particularly significant name *Phantom Tumor.*

I have thus endeavored to condense (see Ranking's Abstract for 1855) perhaps all that is yet reported on the subject, before I give any cases of my own, that the reade , who may not have turned his attention this way, may, without further labor, have a compendious view of the whole matter. And while I frankly acknowledge that these Phantom Tumors have never assumed so grave and illusory an aspect in my view, as they appear to have done in the eyes of some of our European brethren, it may be, my future experience may make it otherwise.

After premising the following instructive quotation, I shall give a few cases of these whimsical conglobations from my own practice, and dismiss the subject for the present.

"The chief importance of the case is in the lesson they convey, as to the necessity of great caution, before pronouncing positively, as to the existence of an abdominal tumor. The surgeon should always be content, in doubtful cases, to examine his patient, on several separate occasions, before venturing an opinion. In most cases, probably, the careful employment of percussion and palpitation will be competent to decide the question correctly; but if there should be the least doubt remaining, the diagnosis should be deferred, until after the free action of a purgative, a second examination has been instituted."

The most interesting case of Phantom Tumor which I have in my Memoranda, occurred in a married woman of about 24 years of age. Her husband left her, a consumptive, to remain in California, for a time, in order to improve his health. He afterward tried Oregon; and finally sailed to the Sandwich Isles, and died there. Before his death (perhaps a year) his wife (whose kindred evinced no scrofulous taint) was frequently troubled with hemoptysis, especially after exertion; a cough soon followed, attended with emaciation and other symptoms of an alarming nature; and these, together with her anxiety and grief for her absent companion, laid the foundation for serious nervous disturbances, displaying themselves in various and singular hysterical paroxysms.

Meanwhile scrofulous tumors began to appear in the cervical region; and ultimately augmenting in number and size, they occupied the greater part of the neck. It was during the progress of this state of things, as we were occasionally counting the tumors one by one as they appeared, that she said to me one day: "Doctor, these lumps are beginning to appear in my bowels now."

I attempted to explain to her the nature and origin of these parabysmic affections as I naturally supposed them to be; but (to borrow a seaman's phrase) I was taken aback, soon afterward, by her querying, why it was, that when she rubbed these "lumps," they would disappear, and then come back again. Sometimes, said she, they seem as if they were floating in water; and when I press on them, they glide away from under my hand, and I can't find them again for some time.

My own curiosity was now excited, and I proceeded to make a manual examination of the tumors; and found her description to be strikingly accurate. All that could be determined was, that they were smooth, and apparently elastic; for the moment an effort was made to define their boundaries and determine their consistence, by grasping the masses with the fingers, they would vanish with a kind of vermicular motion. The tumors were in the right hypochondriac and epigastric regions.

The foregoing traits, and the results of percussion, readily satisfied me that I was wrong in my first explanation, founded wholly on conjecture. Taking in view her hysterical habit, I now told her, I was glad to find that this feature in her case, was of a comparitively trivial character, as I hoped the results of the treatment would show. I conceived that partial spasmodic contractions took place in the intestinal canal, from the presence of air and other exciting bodies, and that handling the parts, increased the peristaltic action of the intestine, for the time being, and caused the tumor to disappear.

I gaver her a cathartic dose of

Rhubarb 5 grs.
Ipecac. 1 gr.
Ginger 3 grs.

After the action of this laxative, I advised:

Tinct. of Galbanum ℥i.

to be repeated every 6 hours, during the day. She did not continue the tincture long, for the illusory tumors soon disappeared, and never returned.

Although I do not consider the term Phantom Tumor, so appropriate, in the next cases to be given, as it is of that class of which a specimen has just been described; yet, as similar instances have been arranged under this head by a London writer, I shall relate them, by way of variety, and in the hope that they may be cautions to the young practitioner.

In the early part of my professional career, a medical friend requested me to visit an "obstetrical case" in consultation with him. He stated that labor had commenced five days before; the pains were strong and regular, and indeed so severe, that the patient thought she could not endure them much longer; that, (his fingers being short,) he could barely reach the os uteri; and that he thought there was no dilatation, after the pain. His patience was exhausted in wearisome waiting. The woman (who had never had children), said that she had gone more than a month beyond her time; and some of her female friends in attendance, corroborated the probability of her calculation, by reference to a biblical case of ten months gestation.

I found the usual pregnant rotundity of the abdomen; the cessation of the catamenia; regular and violent expulsive efforts; and bowels opened daily. There was clearly no dilatation of the os uteri. The case was perplexing.

Kergaradec's "Memoir sur l'Auscultation appliqué a l'etude de la grossesse" had not been published many years; but instructed in his method of detecting advanced pregnancy I had applied the stethoscope, by way of information, in a few cases, and was glad of the present opportunity of again testing its value.

After auscultating in the most thorough manner I was capable of, I could detect no fetal pulsations and no placental sounds. I therefore, rather rashly, but (fortunately for my credit, as it proved), correctly, declared that she *was not pregnant*.

Happily, also, I had read not long before of some cases in Ireland, of vast fetal accumulation, notwithstanding the rectum was emptied every day. I therefore proposed to the doctor, to empty the bowels by a thorough-going cathartic, and see what was in them. He readily assented. The dose was given; volumes of wind and abundance of fecal matter were expelled; the abdomen shrunk, the pains ceased, and to the mortification of the would-be mother, the surprise of the doctor, and my own gratification, the *child was not there*.

This case may remind the reader of Prescott's Philipe Second, of the pseudo-pregnancy of the English Queen Mary, who, however, died dropsical.

As an off-set to my skill in the foregoing instance, I must now relate, though rather out of place, a case which soon after occurred in my own practice. The patient had ceased child-bearing for the last five years, but now believed herself to be in labor. The improbability of her being pregnant, was increased, as I thought, by the os tincæ being nearly out of reach; and flushed with my success in the case just narrated, I again flourished the stethoscope, and found no fetal circulation, no placental sound. "I shouldn't be surprised," said I to those present, "if there is no child here." In the evening, they sent for me in haste and I delivered the woman of a healthy infant, much to the diversion of those who had heard my remark in the morning.

I had intended to detail some other cases of error of diagnosis in abdominal enlargements; but I find I have already written more than I originally designed to; and therefore submit the foregoing, without addition, to the young practitioners of the West.—*Medical Counselor.*

